# Chromosome-level assembly reveals extensive rearrangement in saker falcon and budgerigar, but not ostrich, genomes

**DOI:** 10.1186/s13059-018-1550-x

**Published:** 2018-10-24

**Authors:** Rebecca E O’Connor, Marta Farré, Sunitha Joseph, Joana Damas, Lucas Kiazim, Rebecca Jennings, Sophie Bennett, Eden A Slack, Emily Allanson, Denis M Larkin, Darren K Griffin

**Affiliations:** 1 School of Biosciences, University of Kent, Canterbury, UK; 2 Department of Comparative Biomedical Sciences, Royal Veterinary College, University of London, London, UK

**Keywords:** Chromosome-level genome assembly, Genome evolution, CNE, EBR

## Abstract

**Background:**

The number of de novo genome sequence assemblies is increasing exponentially; however, relatively few contain one scaffold/contig per chromosome. Such assemblies are essential for studies of genotype-to-phenotype association, gross genomic evolution, and speciation. Inter-species differences can arise from chromosomal changes fixed during evolution, and we previously hypothesized that a higher fraction of elements under negative selection contributed to avian-specific phenotypes and avian genome organization stability. The objective of this study is to generate chromosome-level assemblies of three avian species (saker falcon, budgerigar, and ostrich) previously reported as karyotypically rearranged compared to most birds. We also test the hypothesis that the density of conserved non-coding elements is associated with the positions of evolutionary breakpoint regions.

**Results:**

We used reference-assisted chromosome assembly, PCR, and lab-based molecular approaches, to generate chromosome-level assemblies of the three species. We mapped inter- and intrachromosomal changes from the avian ancestor, finding no interchromosomal rearrangements in the ostrich genome, despite it being previously described as chromosomally rearranged. We found that the average density of conserved non-coding elements in evolutionary breakpoint regions is significantly reduced. Fission evolutionary breakpoint regions have the lowest conserved non-coding element density, and intrachromomosomal evolutionary breakpoint regions have the highest.

**Conclusions:**

The tools used here can generate inexpensive, efficient chromosome-level assemblies, with > 80% assigned to chromosomes, which is comparable to genomes assembled using high-density physical or genetic mapping. Moreover, conserved non-coding elements are important factors in defining where rearrangements, especially interchromosomal, are fixed during evolution without deleterious effects.

**Electronic supplementary material:**

The online version of this article (10.1186/s13059-018-1550-x) contains supplementary material, which is available to authorized users.

## Background

The number of de novo (new species) genome sequence assemblies is increasing exponentially (e.g., [1, 2]). Improved technologies are generating longer reads, greater read depths, and ultimately assemblies with fewer, longer contigs per genome [3, 4]; however, the ability to assemble a genome with the same number of scaffolds or contigs as chromosomes (“chromosome-level” assembly) remains the ultimate aim of a de novo sequencing effort. This is for several reasons, among them the requirement for an established order of DNA markers as a pre-requisite for revealing genotype-to-phenotype associations for marker-assisted selection and breeding, e.g., in species regularly bred for food production, companionship, or conservation purposes [5].

Chromosome-level assemblies were rapidly established for agricultural animals (chicken, pig, cattle, sheep) [6–9] in part because they were assembled as maps prior to (e.g., Sanger) sequencing. Species used for food consumption in developing countries (e.g., goat, camel, yak, buffalo, ostrich, quail); animals bred for conservation (e.g., falcons and parrots), and companion animals (e.g., pet birds) are still however poorly represented, in part because they were initially assembled using NGS data alone. New techniques, e.g., optical mapping [10], BioNano [11], Dovetail [12], and PacBio long-read sequencing [13], make significant steps towards this. Recent progress on the goat genome for instance resulted in a chromosome-level assembly using PacBio long-read sequencing [2]; others however encounter technical issues: BioNano contigs fail to map across multiple DNA nick site regions, centromeres, or large heterochromatin blocks, and PacBio requires starting material of hundreds of micrograms of high molecular weight DNA, thereby limiting its usage. To achieve a chromosome-level assembly therefore often requires a combination of technologies to integrate the sequence data, e.g., Hi-C [14], linkage mapping, pre-existing chromosome-level reference assemblies, and/or molecular cytogenetics [15, 16]. To this end, we made use of bioinformatic approaches, e.g., the Reference-Assisted Chromosome Assembly (RACA) algorithm [17]. RACA however is limited in needing a closely related reference species for comparison [17] and further mapping of superscaffolds physically to chromosomes. We therefore recently developed an approach where RACA produces sub-chromosome-sized predicted chromosome fragments (PCFs) which are subsequently verified and mapped to chromosomes using molecular methods [15]. In so doing, we previously established a novel, integrated approach that allows de novo assembled genomes to be mapped directly onto the chromosomes of interest and displayed the information in an interactive browser (Evolution Highway) to allow direct, chromosome-level comparison. To date however, only two genomes—the pigeon (*Columba livia*) and the Peregrine falcon (*Falco peregrinus*)*—*have been assembled in this way [15].

In the current study, we focused on generating chromosome-level assemblies for three further avian genomes. These are the following: The saker falcon (*Falco cherrug*—FCH), classified as endangered [18], is phenotypically remarkable for its visual acuity [19] and acceleration speeds [20]. It has an atypical avian genomic structure (2*n* = 52) with fused microchromosomes [21]. Secondly, we selected the common budgerigar (*Melopsittacus undulatus*—MUN) which also has a highly rearranged karyotype with multiple fusions (2*n* = 62). As a member of the order *Psittaciformes* (parrots), the budgerigar is one of the world’s most popular companion animals as well as a highly valued model for studies into vocal learning [22]. Finally, we selected the ostrich (*Struthio camelus*—SCA), the largest extant bipedal land animal [23]. The ostrich is able to travel long distances with a remarkable degree of metabolic economy [24]. Apparently possessing a typical avian karyotype (2*n* = 80), with a large degree of homology with the chicken (like other ratite birds) revealed by cross species chromosome painting [25–27], it however purportedly has 26 previously undetected interchromosomal rearrangements when compared to the ancestral avian karyotype as revealed by sequence assembly analysis of optical mapping data [28]. For these three species, we used our previously described approach combining computational algorithms for ordering scaffolds into predicted chromosome fragments (PCFs) which we then physically mapped directly to the chromosomes of interest using a set of avian universal bacterial artificial chromosome (BAC) probes [15].

Chromosome-level assemblies also inform studies of evolution and speciation given that inter-species differences arise from chromosomal changes fixed during evolution [29–35]. In recent studies, we have used (near) chromosome-level assemblies to reconstruct ancestral karyotypes and trace inter- and intrachromosomal changes that have occurred to generate the karyotypes of extant species [28, 36]. Theories explaining the mechanisms of chromosomal change in vertebrates include a role for repetitive sequences used for non-allelic homologous recombination (NAHR) in evolutionary breakpoint regions (EBRs) [37] and the proximity of DNA regions in chromatin [38]. During gross genome (karyotype) evolution, unstable EBRs delineate stable homologous synteny blocks (HSBs) and we have established that the largest HSBs are maintained non-randomly and highly enriched for conserved non-coding elements (CNEs) [9–11, 15, 39]. We recently proposed the hypothesis that a higher fraction of elements under negative selection involved in gene regulation and chromosome structure in avian genomes (~ 7%) [40] compared to mammals (~ 4%) [41] could contribute to some avian-specific phenotypes, as well as the evolutionary stability of the overall organization of most avian genomes [39]. We further studied the fate of CNEs in the EBRs flanking interchromosomal rearrangements of a highly rearranged avian genome, finding that, in the peregrine falcon, interchromosomal EBRs contain 12 times fewer CNEs than intrachromosomal ones [15].

In order to investigate the role of CNEs in chromosome rearrangements further, we therefore concentrated on species that had previously been reported as highly chromosomally rearranged. Studying these highly rearranged genomes at this resolution provided insight into the mechanisms of chromosomal rearrangement.

## Results

### Predicted chromosome fragments for three new species

Predicted chromosome fragments were generated for fragmented saker falcon, budgerigar, and ostrich whole-genome sequences using RACA [17]. The zebra finch and the chicken chromosome assemblies were used as reference and outgroup respectively for all reconstructions, except for ostrich. For saker falcon, we generated 95 PCFs representing 97.26% of the original genome, while for ostrich and budgerigar, 100 and 84 PCFs were produced (Table 1). These initial PCF sets contained ~ 10% putatively chimeric scaffolds for both ostrich and saker falcon, while for budgerigar, ~ 31% of the scaffolds were split by RACA due to insufficient read and/or comparative evidence to support their structures.

**Table 1:** Statistics for the scaffold split regions tested by PCR

Statistics	Saker falcon	Ostrich	Budgerigar
Pair-end read physical coverage within tested scaffolds	135–524	2–604	0–631
No. split SF adjacencies by RACA (default param.)	61	69	154
No. tested scaffold split regions	22 (100%)	49 (100%)	43 (100%)
No. amplified split regions (confirmed SF joints)	11 (50%)	32 (65%)	20 (46%)
No. non-amplified split regions	11 (50%)	17 (35%)	23 (54%)
No. tested RACA-suggested adjacencies	11	8	18
No. amplified adjacencies (chimeric SF joints)	5	7	11
Final no. ambiguous SF joints from tested split regions	6	10	12
Selected pair-end read spanning threshold	379	239	216

We then tested the split scaffold regions by PCR to assess their existence in the target genome. Only the split regions defined to < 6 kbp in the target genomes were tested, representing 36%, 71%, and 28% of all split scaffolds in the saker falcon, ostrich, and budgerigar assemblies, respectively (Table 1). Of these, 11, 20, and 32 resulted in amplicons of expected length in saker falcon, budgerigar, and ostrich genomic DNA, respectively. For the split regions with negative PCR results, we tested an alternative (RACA-suggested) order of the flanking syntenic fragments (SFs). Out of these, amplicons were obtained for 5/11 in saker falcon, 11/23 in budgerigar, and 7/17 in ostrich, confirming the chimeric nature of the original scaffolds properly detected in these cases. As in our previous publication [15], to estimate which of the remaining split regions (> 6 kb; 39 in falcon, 111 in budgerigar, and 20 in ostrich PCFs) were likely to be chimeric, we empirically identified the genome-wide minimum physical coverage [42] levels for each species in the SF joining regions for which the PCR results were most consistent with original scaffold structures. A physical coverage of 379×, 216×, and 239× were estimated for saker falcon, budgerigar, and ostrich to produce the highest agreement between scaffolds and PCR results. Finally, we used the adjusted physical coverage thresholds to reconstruct a new set of PCFs for all three species (Table 1). To do so, we re-ran RACA by updating the MIN_INTRACOV_PERC parameter with the new physical coverage thresholds (Table 1) and including scaffolds with the structures confirmed by PCR as additional inputs. This resulted in an increased number of PCFs, a reduction of the N50, and a lower fraction of chimeric scaffolds for all species.

### Chromosome-level assemblies for three new species

**Fig. 1:**
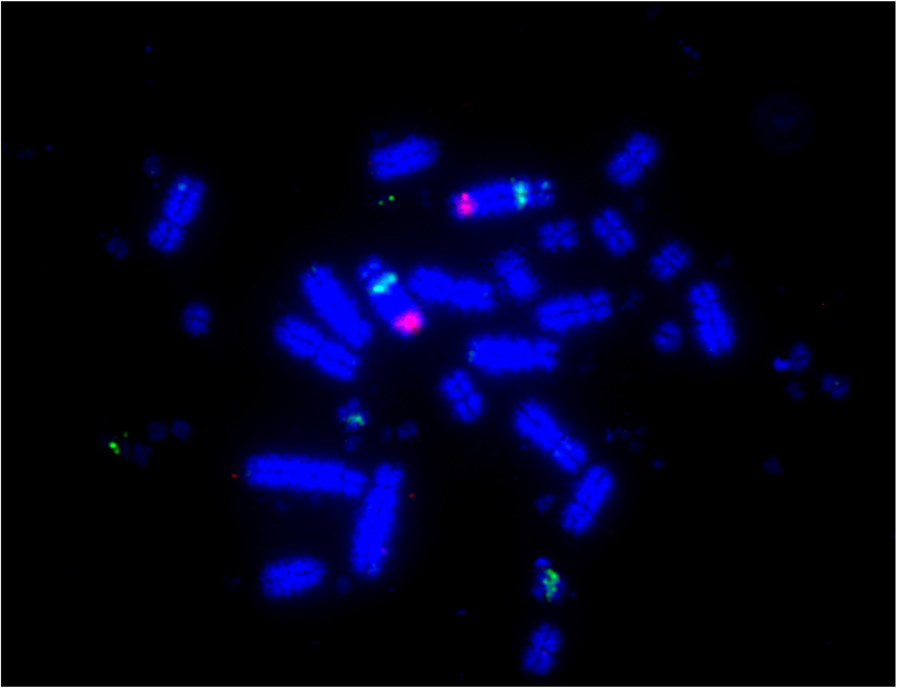
BAC clones hybridized to budgerigar chromosome two (MUN2). The green (FITC labeled) signal represents TGMCBA-375I5 (GGA17 homolog) and maps to PCF 17, and the Texas red labeled signal represents CH261-169K18 (GGA3 homolog) and maps to PCF 3c_5a

**Fig. 2:**
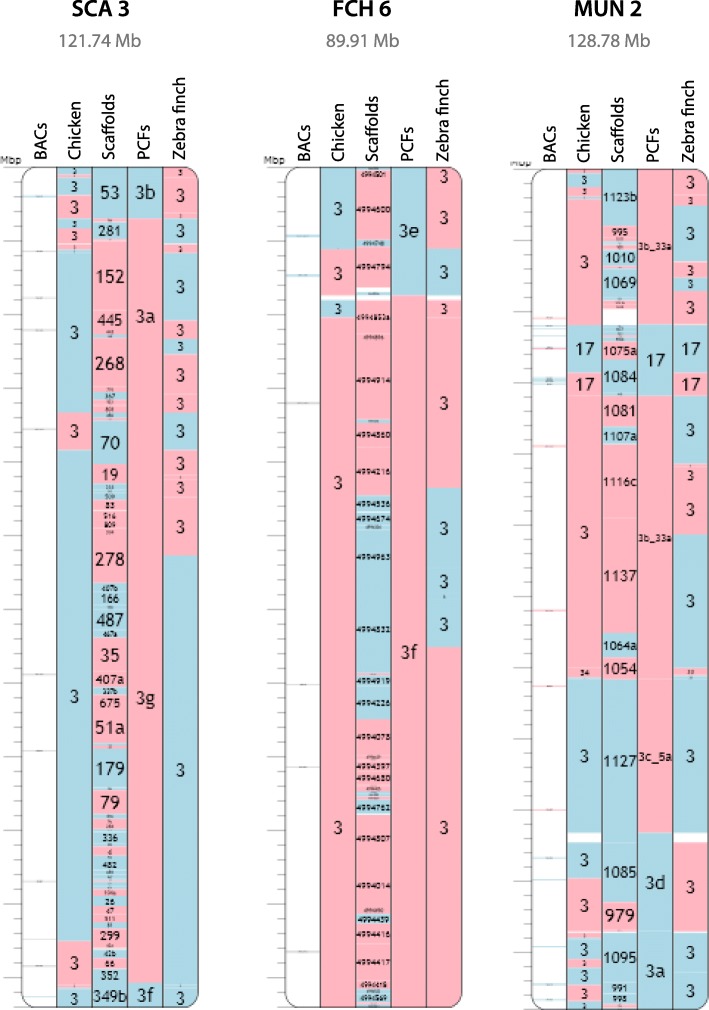
Chromosomes homologous to chicken (ancestral) chromosome 3 with mapped BACs, scaffolds, PCFs, and zebra finch homologies shown. SCA3 = ostrich chromosome 3, FCH6 = saker falcon chromosome 6, MUN6 = budgerigar chromosome 2. The full dataset can be found on the interactive browser Evolution Highway at the following link: http://eh-demo.ncsa.uiuc.edu/birds/

We successfully generated chromosome-level assemblies for the three avian species of interest, with coverage similar to Sanger sequencing assembled genomes. Our method involves (a) construction of PCFs for fragmented assemblies based on the comparative and sequence read data implemented in the RACA algorithm, (b) PCR and computational verification of a limited number of scaffolds that are essential for revealing species-specific chromosome structures, (c) creation of a refined set of PCFs using the verified scaffolds and adjusted adjacency thresholds in RACA, and (d) the use of a panel of “universal” BAC clones to anchor PCFs to chromosomes in a high-throughput manner (see Fig. 1 for representative image) and is reported in detail elsewhere [15]. Using this approach, for the ostrich (2*n* = 80), the N50 of the original NGS genome was improved approximately eightfold, with over 79% of the genome placed onto chromosomes with 71.26% of the original assembly fully oriented (see Table 2). Chromosome-level assembly was accomplished for all GGA (chicken) homologs with the exception of chromosome GGA16 for which BAC clones were not available. PCFs were generated ranging in size from 350 kb to 82 Mb; the second largest of which (80.5 Mb) represented the entire p-arm of chromosome 1. For the budgerigar (2*n* = 62), FISH mapping (e.g., Fig. 1) resulted in 21 pairs of budgerigar autosomes and the Z chromosome being assembled with a fourfold improvement on the scaffold N50 from 11 to 38 Mb. 93.56% of the original assembly was placed onto chromosomes, and 77.93% was fully oriented. For the Saker falcon (2*n* = 52), in total, 19 autosomes and the Z chromosome were assembled to chromosome level, with a fivefold N50 improvement, resulting in 90.12% of the original assembly assigned to chromosomes and 67.52% of the assembly fully oriented. Assembly statistics for all three genomes are listed in Table 2. In the course of the FISH experiments performed, we did not detect any BAC spanning breakpoints. A representative screenshot (Fig. 2) of chromosomes homologous to ancestral chromosome 3 is given (BACs, scaffolds, and PCFs shown), and the whole dataset is freely available on http://eh-demo.ncsa.uiuc.edu/birds/.

**Table 2:** Assembly statistics from original NGS genome to RACA assembly and combined RACA and FISH assembly

Original assembly			
Stats	Budgie	Ostrich	Saker falcon
No. scaffolds longer 10 kbp	1138	1179	731
Total length (Gbp)	1.08	1.22	1.17
N50 (Mbp)	11.41	3.64	4.16
Default RACA assembly			
Stats	Budgerigar PCFs	Ostrich PCFs	Saker falcon PCFs
No. PCFs	84	100	95
Total length (Gbp)	1.04	1.17	1.14
N50 (Mbp)	46.54	37.95	39.38
No. chimeric scaffolds	80 (31%)	58 (10%)	50 (10%)
No. used scaffolds	254	588	458
% original assembly	96.29	95.90	97.26
RACA + PCR assembly			
Stats	Budgerigar PCFs	Ostrich PCFs	Saker falcon PCFs
No. PCFs	95	136	103
Total length (Gbp)	1.04	1.17	1.14
N50 (Mbp)	37.96	28.09	22.28
No. chimeric scaffolds	55 (21%)	31 (5%)	25 (5%)
No. used scaffolds	254	588	458
% original assembly	96.29	96.02	97.26
RACA + FISH assembly			
Stats	Budgerigar chromosomes	Ostrich chromosomes	Saker falcon chromosomes
No. PCFs placed	46	53	64
No. PCFs oriented	28	37	37
Disagreements RACA-FISH	4	0	0
Length placed (bp)	1,013,720,408	969,537,146	1,055,312,481
Length oriented (bp)	844,433,024	869,521,333	790,725,803
% original assembly placed	93.56	79.45	90.12
% original assembly oriented	77.93	71.26	67.52

### Comparative genomics with chicken

All three species were aligned against the chicken (*Gallus gallus—*GGA) genome assembly. Chicken is the most characterized avian genome at sequence depth and chromosome level [6], and the species considered to be most similar chromosomally to the avian ancestor [28].

**Fig. 3:**
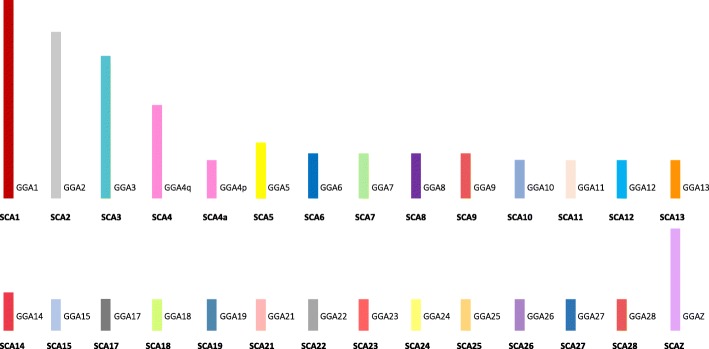
Ideogram representation of the gross genomic structure of the ostrich (*Struthio camelus*—SCA) with chicken homologies per chromosome. Each GGA (chicken) homolog is represented as a different color—randomly assigned. Intrachromosomal differences are not shown here but listed in Additional file 1: Table S1

Homology between the ostrich and the chicken (as illustrated in Fig. 3) was confirmed interchromosomally between all chromosomes tested, with the exception of GGA4 which is homologous to ostrich chromosome 4 *plus* one microchromosome (a fusion thought to have occurred in the chicken lineage [43]). Contrary to our previous study [28], we found no further evidence of interchromosomal rearrangement compared to the chicken. A total of 14 intrachromosomal differences were identified in the ostrich when compared to the chicken listed in Additional file 1: Table S1.

**Fig. 4:**
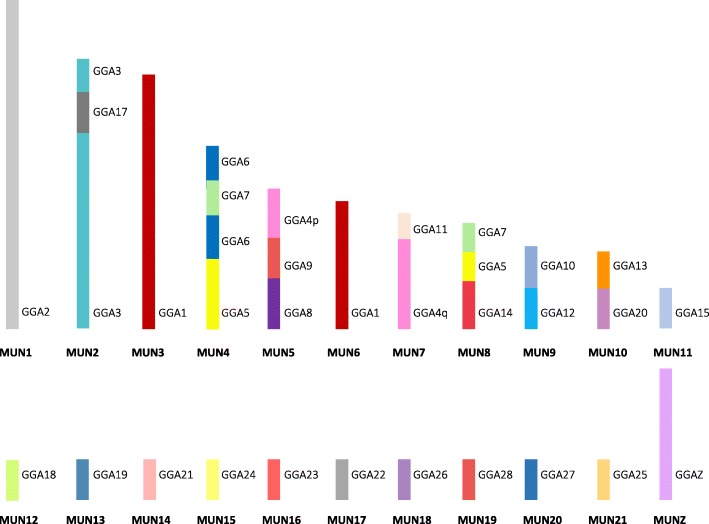
Ideogram representation of the gross genomic structure of the budgerigar (*Melopsittacus undulatus*—MUN) with chicken homologies per chromosome. Each GGA (chicken) homolog is represented as a different color as assigned in Fig. 3. Intrachromosomal differences are not shown here but listed Additional file 1: Table S2

Homologies between the budgerigar and the chicken were identified for all mapped chicken chromosomes (GGA1-28, excluding 16, plus Z). Fusions of ten homologs were identified with three budgerigar chromosomes (MUN4, 5, and 8), exhibiting the fusion of three chicken homologs each (Fig. 4). The fusion of two chicken homologs was demonstrated in three budgerigar chromosomes (MUN2, 9, and 10). Three fissions were evident where the GGA1 homolog split to form MUN3 and 6 with no evidence of further fusion; GGA5 and GGA7 homologs split and fused as separate chromosomes (MUN4 and 8). The GGA4 homolog exhibited the pattern seen in most other birds where the p-arm of GGA4 is in fact a fused ancestral microchromosome. Where previously assigned, the budgerigar chromosomes were numbered according to Nanda et al. [44]. Where no previous assignment had been given, the chromosomes were numbered according to decreasing PCF size. A representative ideogram illustrating the gross genomic structure and the chicken homologies is shown in Fig. 4. In total, of the 18 mapped chicken microchromosome homologs, 7 were fused to other chromosomes, while 11 remained intact as microchromosomes. Given the deviation from the typical avian pattern, these interchromosomal changes are thought to be unique to the budgerigar lineage. A total of 16 intrachromosomal rearrangements were identified between budgerigar and chicken, none of which were seen in the ostrich-chicken comparison, nor in the 14 chicken-specific intrachromosomal changes reported by Farre et al. [39], suggesting that these arose after the Galloanserae-Neoaves divergence (illustrated in Additional file 1: Table S2).

**Fig. 5:**
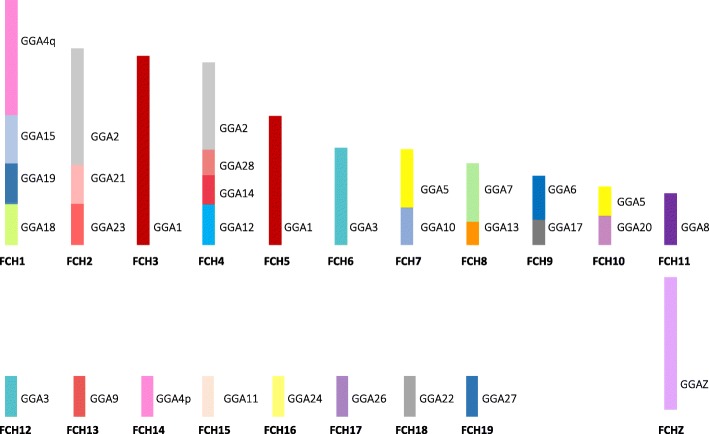
Ideogram representation of the gross genomic structure of the saker falcon (*Falco cherrug*—FCH) with chicken homologies per chromosome. Each GGA (chicken) homolog is represented as a different color as assigned in Fig. 3. Intrachromosomal differences are not shown here but listed in Additional file 1: Table S3

Extensive interchromosomal genome rearrangement was evident in the saker falcon where, in total, 12 fusions and 5 fissions were detected when compared to the chicken genome. Each of the largest chicken macrochromosome homologs (GGA1 to GGA5) were represented by two saker falcon chromosomes indicating fission in the falcon lineage for chromosomes 1, 2, 3, and 5 but the commonly reported chicken lineage fusion for GGA4. Both the GGA6 and GGA7 homologs were found as single blocks fused with other chicken homologs while GGA8, GGA9, and GGAZ were represented as individual chromosomes. Of the 17 mapped chicken microchromosomes, regions homologous to GGA microchromosomes 10, 12, 13, 14, 15, 17, 18, 19, 20, 21, 23, and 28 were fused to GGA macrochromosome homologs, leaving GGA 11, 22, 24, 26, and 27 conserved as intact microchromosomes. The overall genomic structure is illustrated in Fig. 5, with saker falcon chromosomes numbered according to size. A total of 36 intrachromosomal differences were identified when compared to the chicken, none of which were evident in the ostrich-chicken comparison, nor in the 14 chicken-specific intrachromosomal changes reported by Farre et al. [39], suggesting that these are probably unique to the falcon lineage, arising after the Galloanserae-Neoaves divergence. These are illustrated in Additional file 1: Table S3.

### Rearrangements from the avian ancestor

**Fig. 6:**
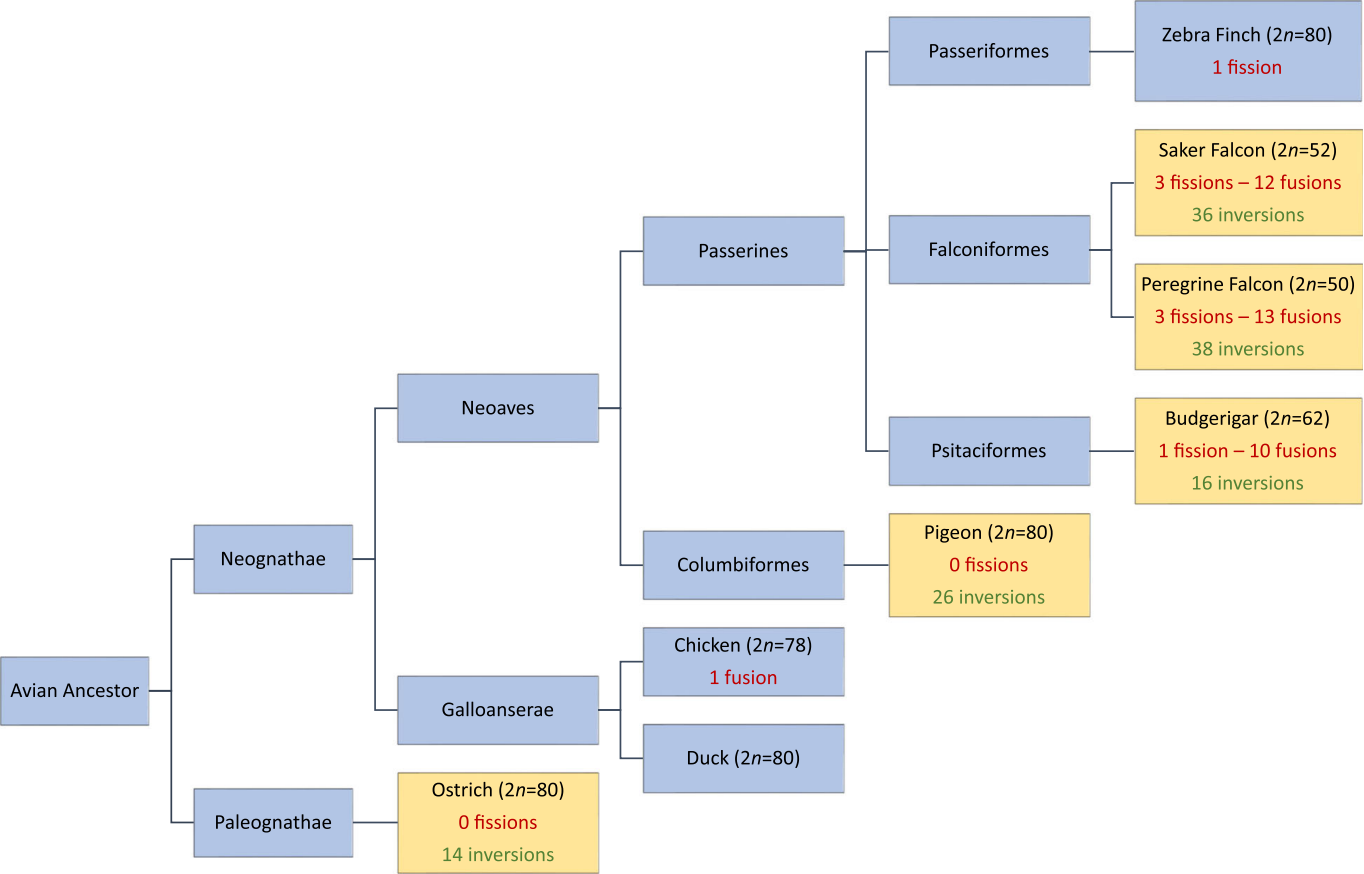
Phylogenetic tree highlighting the relationship of species analyzed here and in our previous study [15] demonstrating the number of inter- and intrachromosomal rearrangements that have occurred relative to the avian ancestor (interchromosomal) and the chicken (intrachromosomal). Species investigated using our approach are highlighted in yellow with other species (chicken, duck, and zebra finch) represented for context. Phylogeny is based on Burleigh et al. [70]

The overall pattern of chromosomal rearrangement evident in the three species is illustrated in Table 3 and Fig. 6 by divergence from the inferred avian ancestor. Given the similarity interchromosomally of chicken and ostrich, and the prior knowledge that GGA4 arose from the fusion of two ancestral chromosomes, the single interchromosomal difference (GGA4 fusion) is easily derived. For the intrachromosomal changes, using ostrich as an outgroup infers the changes since the divergence of the Neognathe ancestor (see above). In the absence of a chromosomally assembled outgroup genome for all birds in this study, it is not easy to determine whether the intrachromosomal differences are ancestral or derived in chicken and ostrich respectively. For this reason, in the far-right hand column of Table 3, the differences between chicken and ostrich are noted but without any conclusions as to which is the ancestor.

**Table 3:** Patterns of fusion and fission revealed in the budgerigar, saker falcon, and the ostrich using the chicken genome as a reference

Ancestral chromosome (numbered according to chicken)	Budgerigar		Saker falcon		Ostrich	Chicken	Chicken-ostrich differences
	Inter-	Intra-	Inter-	Intra-	Inter-	Inter-	Intra-
1	Fission	1	Fission	4	0	0	0
2	–	2	Fission and fusion to GGA21 and 28	0	0	0	0
3	Fusion to GGA17	3	Fission	4	0	0	3
4a	–	0	–	2	0	Fusion	1
4b	Fusion to GGA9	0	Fusion to GGA15	4	0		2
5	Fission and fusion to GGA6	1	Fission and fusion to GGA10 and 20	2	0	0	1
6	Fusion to GGA5	0	Fusion to GGA17	3	0	0	0
7	Fission and fusion to GGA6 and 5	1	Fusion to GGA13	2	0	0	3
8	Fusion to GGA9	0	–	0	0	0	1
9	Fusion to GGA8	1	–	0	0	0	0
10	Fusion to GGA12	0	Fusion to GGA5	0	0	0	0
11	Fusion to GGA4q	0	–	0	0	0	0
12	Fusion to GGA10	1	Fusion to GGA14	0	0	0	0
13	Fusion to GGA20	0	Fusion to GGA7	0	0	0	0
14	Fusion to GGA5	1	Fusion to GGA12 and 28	1	0	0	1
15	–	2	Fusion to GGA4q and 19	2	0	0	0
16	No data	No data	No data	No data	No data	No data	No data
17	Fusion to GGA3	0	Fusion to GGA6	1	0	0	0
18	–	1	Fusion to GGA19	2	0	0	1
19	–	0	Fusion to GGA15 and 18	0	0	0	0
20	Fusion to GGA13	0	Fusion to GGA5	0	0	0	0
21	–	0	Fusion to GGA2 and 23	0	0	0	0
22	–	0	–	2	0	0	1
23	–	2	Fusion to GGA21	2	0	0	0
24	–	0	–	1	0	0	0
25	No data	No data	No data	No data	No data	No data	No data
26	–	0	–	2	0	0	0
27	–	0	–	1	0	0	0
28	–	0	Fusion to GGA2 and 14	1	0	0	0
Z	–	0	–	0	0	0	0

The left-hand column represents the ancestral avian chromosome, with the subsequent columns indicating the number of inter- and intrachromosomal changes detected that have led to each extant species. For the intrachromosomal differences between ostrich and chicken, in the absence of an outgroup, the direction of change cannot be determined and thus only differences between the two species is noted

There were two fissions common to both the budgerigar and the saker falcon. The first of these involved the chicken chromosome 1 homolog (FCH3 and 5; MUN3 and 6) where the fission point (between GGA ~ 72 and ~ 86 Mb) corresponds to the breakpoint seen in the chromosomally assembled zebra finch genome (between GGA ~ 74 and ~ 75 Mb), and probably in all Passerines according to zoo-FISH studies [45]. The second was a fission that occurred in the homolog of chicken chromosome 5, the derivative products of which went on to form budgerigar chromosomes 4 and 8 and saker chromosomes 7 and 10. Finally, a fission present in falcon but not in budgerigar (chromosome 2 centric) is also observed in turkey, but is probably an example of homoplasy given that centromeres are prone to fission.

In the budgerigar genome, 13 chicken homologs showed no evidence of fission or fusion and in the saker, 8 homologs showed no evidence of interchromosomal rearrangement. The Z chromosome was the only macrochromosome that did not rearrange interchromosomally in all species tested.

The ostrich was revealed to have the lowest number of intrachromosomal differences relative to chicken, with a total of 14 identified—three of which were on chromosome 3. The budgerigar, although highly rearranged interchromosomally, appeared to have a similar number of intrachromosomal rearrangements, with evidence of 16 inversions, 3 of which were on the homolog of GGA3 (albeit different from those seen in the ostrich). The saker falcon, however, while also highly rearranged at an interchromosomal level, also exhibited a very large number of intrachromosomal changes relative to the other species with 36 inversions. No intrachromosomal rearrangement was evident in the homologs of chromosomes 19, 21, and 25 (Additional file 2).

### CNEs in avian inter- and intrachromosomal EBRs

Analysis of the three new avian genomes, previously thought to have undergone significant interchromosomal rearrangement compared to most avian genomes, allowed us to investigate the role of conserved non-coding elements (CNEs) in inter- vs intrachromosomal rearrangement. Our results determined that only two genomes were in fact highly rearranged interchromosomally. A total of 27 inter- and 146 intrachromosomal EBRs were identified in the three genomes (listed in Additional file 3: Tables S7–S9). We calculated densities of CNEs [39] in both types of EBRs using chicken genome as a reference. Intra- and interchromosomal EBRs were defined to ≤ 100 kb in the chicken genome. Avian EBRs had a significantly lower fraction of CNEs than their two adjacent chromosome intervals of the same size each (up- and downstream; *P* = 0.01). Moreover, the interchromosomal EBRs (fusions and fissions) had, on average, approximately 2.2 times lower density of CNEs than the intrachromosomal EBRs (*P* = 2.40 × 10^–5^). The lowest density of CNEs was observed in the fission breakpoints (*P* = 0.04). In order to identify the CNE densities and the distribution associated with avian EBRs at the genome-wide level, we further counted CNE bases in 1-kb windows overlapping EBRs and avian multi-species HSBs (msHSBs) > 1.5 Mb [39]. The genome-wide CNE density was 0.087, close to the density observed in msHSBs. The average density of CNEs in the EBR windows was significantly lower (0.022) than that in the msHSBs (0.107, *P* < 0.01). Fission EBRs had the lowest density of CNEs observed, approximately zero CNE bases, while in the intrachromomosomal, EBRs had the highest among the EBR regions (0.026, *P* = 0.035, Table 4).

**Table 4:** Statistics for CNE density in 1-kb windows for avian EBRs, msHSBs, and genomewide

	Average no. CNE bases	Average density of CNE bases
Genome	86.85	0.087
msHSB	106.81	0.107
Intra	26.14	0.026
Fusion	13.76	0.013
Fission	5.48	0.005
EBR*	23.76	0.024

*Fission, fusion, and intrachromosomal EBRs combined

## Discussion

Increasing numbers of newly sequenced genomes require tools that facilitate inexpensive, efficient chromosome-level assembly for the reasons described above. The tools used here and developed in our previous study [15] have generated chromosome-level assemblies for previously published but highly fragmented sequenced genomes. The assemblies generated using this approach now have > 80% of their genomes placed on chromosomes, making them highly comparable to genomes assembled using Sanger sequencing techniques and high-density physical or genetic mapping [29]. The method used here is less expensive and requires fewer resources than pre-existing approaches, in part thanks to the ability to generate predicted chromosome fragments of a sub-chromosomal size using comparative genome and not end existing read pair information only. The subsequent use of BAC probes designed to work equally well on a large number of highly diverged avian species creates a resource for physical mapping that is transferrable potentially to all avian species.

### The ostrich genome

Avian interchromosomal rearrangements are rare, except in cases (e.g., *Psittaciformes* and *Falconiformes*) where it is evident that karyotypes are highly rearranged [15, 45, 46]. In the case of the ostrich and other ratites (emu and rhea), avian-typical patterns have been illustrated using comparative chromosome painting [25–27]. However, results presented in our previous study [28], based on NGS assemblies enhanced with newer third-generation technologies, suggested that the ostrich is in fact the exception to this pattern. Our older data that included optical map-enhanced NGS assembly [47] indicated the presence of 26 ostrich interchromosomal rearrangements compared to the avian ancestor. The data presented in the current study however contradicts these findings and confirms the original chromosome painting data that the ostrich genome is in fact a “typical” avian genome in terms of overall karyotypic structure. The most likely explanation for these erroneously called interchromosomal rearrangements is errors in either the optical map data itself or the original Illumina scaffolds that were enhanced by the map, again, highlighting the importance of anchoring genome sequences to the chromosomes directly, rather than relying purely on a sequence-based and single mapping approach. In this regard, therefore, our previous results generated somewhat of a paradox in that ostrich molecular branch lengths appeared short but the ostrich “genome rearrangement branch length” appeared relatively long. The results presented here however resolve this paradox by providing a new assembly in which there are fewer rearrangements in the ostrich genome.

### The saker falcon and the budgerigar genomes

Among the *Psittaciformes* and *Falconiformes*, few studies of karyotype structure have been performed. Only one zoo-FISH study for each order [21, 44] has attempted to characterize the overall genome structure, finding the limited success common to most zoo-FISH studies. The chromosome painting study on the falcons revealed similar patterns of rearrangement between the peregrine falcon and the common kestrel (*Falco tinnunculus*) (2*n* = 52), but less similarity in the merlin (*Falco columbarius*) (2*n* = 40). The study focusing on *Psittaciformes* revealed patterns of similarity between the budgerigar, the cockatiel, and the peach-faced lovebird [44]. Common to both of these studies was a pattern of rearrangement that was similar among closely related species within the same order; however, when comparing the orders against each other, there were few parallels between them. In both studies, a lack of available tools capable of detecting the microchromosomes in the genome reorganization meant that results were limited to patterns involving the macrochromosomes only. Conversely, results presented here reveal previously undetected rearrangements involving microchromosomes, demonstrating that fusion is the most common mechanism of interchromosomal rearrangement, i.e., there was no evidence of reciprocal translocation. In some examples, particularly in the falcon genomes, multiple microchromosomes have fused together, but have still remained intact as discrete regions of conserved synteny, albeit fused to larger chromosomes. Also revealed through the chromosomal assembly of these genomes is a common breakpoint in the homolog of GGA1. Occurring in the same genomic region in both the budgerigar and the saker falcon, this breakpoint also occurs in the same region of the closely related zebra finch genome suggesting that this occurred in the Australavian ancestor of all three birds (zebra finch, falcon, and budgerigar) and was therefore already fixed in these three descendant lineages.

### Intrachromosomal rearrangements

A comparison of the number of intrachromosomal rearrangements between the species tested here and those assembled in our previous study [15] revealed that the fewest changes (when compared to the chicken genome as the reference) occurred in the ostrich, with evidence of only 14 inversions across the karyotype. Sharing a common ancestor over 100 mya [48], both the ostrich and the chicken are considered to be the most ancestral extant representatives of modern birds. These results suggest that their genomes also exhibit this ancestral pattern with little change between the two species. At the other extreme, the saker falcon examined here and peregrine falcon described previously [15] both exhibit a remarkably large number of changes (with an average of 37 inversions) consistent with the highly rearranged nature of the falcon genome. Surprisingly however, the budgerigar exhibits only 16 intrachromosomal rearrangements (similar to ostrich), suggesting that (unlike the falcons) the chromosomal rearrangement is limited to a pattern of overall interchromosomal change that once fixed, changed relatively little intrachromosomally. The difference between the number of inversions seen in the falcons and the budgerigar is surprising given that they have both been subject to so much chromosomal change. It may be that there is some biological advantage to this gross genomic structure in falcons that does not offer a selective advantage to the parrots, perhaps due to the high metabolic demands required by birds of prey.

Most studies into EBRs and HSBs have focused on mammals, many of which illustrate that EBRs tend to appear in gene-dense regions [49]. These “EBR genes” appear to be related to biological features specific to individual lineages [7, 8, 49]. A pattern of EBR reuse is also evident with some regions of the genome being particularly prone to chromosomal breakage [50, 51]. In fact, among birds (chicken, turkey, and zebra finch), it appears that breakpoint reuse occurs more often than is seen in mammals [43, 52], with previous data produced (comparing chicken and zebra finch) suggesting a key role for recombination-based mechanisms in the generation of chromosome rearrangements [53]. Larkin and colleagues argue that the presence of HSBs across multiple species is the result of a selective advantage to keeping particular combinations of genes together [49], with evidence of gene ontology enrichment for terms related to organismal development and the central nervous system, although some authors refute the notion that these proximity patterns occur or that there is any adaptive significance when they do (e.g., [54, 55]).

Here, however, we focus our studies on CNE distribution, indicating that CNEs are more depleted in EBRs generally but particularly in interchromosomal rearrangements—especially fission. Compared to our previous study [15] based on one interchromosomally rearranged genome (peregrine falcon), in this study, we used two additional genomes including one of which is phylogenetically distant from the peregrine falcon—the budgerigar. Our findings are, however, in line with what we found previously, demonstrating that in avian genomes the CNEs are important factors defining where rearrangements (especially the interchromosomal ones) are able to be fixed in evolution without leading to deleterious effects. This is further reinforced by the fact that chromosomal fissions in both studies are associated with genome intervals having no CNEs at all.

Species that exhibit a high degree of interchromosomal rearrangement (mammals, non-avian reptiles, and amphibians) all tend to have large, repeat-rich genomes that appear to correlate with a higher rate of rearrangement. The results presented here suggest that some avian lineages (such as the falcons and the parrots) also undergo a similar degree of chromosomal change but without the correspondingly large, repeat-rich genome. Instead, comparisons of the zebra finch and the budgerigar suggest that the high chromosomal mutation rates seen in both lineages may in fact be changes that have occurred in response to the exploitation of evolutionary niches, which ultimately end in fixed interchromosomal rearrangements. In the majority of other bird species however, it appears that such fixation is prevented, resulting in maintenance of an overall stable avian karyotype. A large number of CNEs in avian chromosomes (about twice as high as in the mammalian genomes) could form regulatory networks that cannot be altered, contributing to stability of chromosomes.

Why some rearrangements become fixed, and others do not, is a relatively understudied field, although clues may lie in the study of gene ontology terms present in EBRs. Farré and colleagues found a correlation between EBRs and specific avian adaptive features in individual species, including forebrain development in the budgerigar (one of the species investigated here), consistent with this species being not only a vocal learner but having distinctive neuronal connections compared to other vocal learners [39]. As more genomes become available with better assemblies, these analyses may well point to adaptive phenotypic features of individual orders and families.

## Conclusions

By combining comparative sequence analysis, targeted PCR, and high-throughput molecular cytogenetics, the results presented here provide further evidence for an approach that is theoretically applicable to any animal genome as a cost-effective means of transforming fragmented scaffold-level assemblies to chromosomal level. The N50 of each genome was improved significantly, and a series of intra- and interchromosomal rearrangements that were previously undetectable were identified. Most bird genomes remain remarkably conserved in terms of their chromosome number (in 60–70% of species 2*n* = ~ 80) [43, 45, 46, 53], and interchromosomal changes are relatively rare, but when they do occur, they tend to be lineage specific, e.g., in *Psittaciformes* (parrots), *Falconiformes* (falcons), and *Sphenisciformes* (penguins) [15, 45, 56]. Fusion is the most common mechanism of change, there is no evidence yet of reciprocal translocation, and all microchromosomes remain “intact,” even when fused to larger chromosomes. Why some groups exhibit a high degree of interchromosomal rearrangement remains unclear; some (e.g., kingfishers) have an unusually high (2*n* = 130+) number and both higher and lower deviations from the typical (2*n* = ~ 80) organization can occur in the same group. For instance, the Adélie penguin (2*n* = 96) and the emperor penguin (2*n* = 72) suggest that similar mechanisms can cause both a rapid reduction and a rapid increase in chromosome number. The short time period over which these changes occur in the penguins and the rearranged karyotypes of the *Falconiformes* and the *Psittaciformes* (but not the sister group, the *Passeriformes*) suggest that these changes can happen quickly. Vertebrates with large, repeat-rich genomes (such as mammals and amphibians) frequently demonstrate rapid intra- and interchromosomal rearrangements [31]. The results presented here suggest that birds too can undergo similar changes in certain groups although there is little evidence that these highly rearranged avian genomes are particularly large or more repeat rich than other avian genomes.

## Methods

### Avian genome assemblies, repeat masking, and gene annotations

The chicken (Gallus_gallus 4.0 [6]) and zebra finch (WUGSC 3.2.4 [57]) chromosome assemblies were downloaded from the UCSC Genome Browser [58]. The assemblies of saker falcon ostrich and budgerigar were provided by the Avian Phylogenomics Consortium [59]. All sequences were repeat-masked using Window Masker [60] with *-sdust* option and Tandem Repeats Finder [61]. Chicken gene (version of 27/04/2014) and repetitive sequence (version of 11/06/2012) annotations were downloaded from the UCSC Genome Browser [62]. Chicken genes with a single ortholog in the human genome were extracted from Ensembl Biomart (v.74 [63]).

### Pairwise and multiple genome alignments, nucleotide evolutionary conservation scores, and conserved elements

Pairwise alignments using chicken and zebra finch chromosome assemblies as references and other assemblies as targets were generated with *LastZ* (v.1.02.00; [64]) and converted into the UCSC “chains” and “nets” alignment formats with the Kent-library tools ([58]). Conserved non-coding elements obtained from the alignments of 48 avian genomes were used [39].

### Reference-assisted chromosome assembly of avian genomes

Saker falcon, budgerigar, and ostrich PCFs were generated using the Reference-Assisted Chromosome Assembly (RACA [17]) tool. We chose the zebra finch genome as reference and chicken as outgroup for the saker falcon and the budgerigar based on the phylogenetic distances between the species [65]. For the ostrich, we used chicken as the reference and zebra finch as outgroup and vice versa experiments were performed as the ostrich is phylogenetically equally distant from chicken and zebra finch. Two rounds of RACA were done for both species. The initial run was performed using the following parameters: *WINDOWSIZE=10 RESOLUTION=150000 MIN_INTRACOV_PERC=5*. Prior to the second run of RACA, we tested the scaffold split during the initial RACA run using PCR amplification across the split intervals (see below) and adjusted the parameters accordingly as previously described [15].

### PCR testing of adjacent SFs

Primers flanking split SF joints within scaffolds or RACA-predicted adjacencies were designed using Primer3 software (v.2.3.6 [66]). To avoid misidentification of EBRs or chimeric joints, we selected primers only within the sequences that had high-quality alignments between the target and reference genomes and found in adjacent SFs. Due to alignment and SF detection settings, some of the intervals between adjacent SFs could be > 6 kb and primers could not be chosen for a reliable PCR amplification. Whole blood was collected aseptically from adult saker falcon, ostrich, and budgerigar. DNA was isolated using DNeasy Blood and Tissue Kit (Qiagen) following standard protocols. PCR amplification was performed according to the protocol described in [15]. Briefly, PCR amplification was performed in a total volume of 10 μL as follows: 5 μL of DreamTaq Master Mix (Fermentas), 1 μL of each primer at 2 μM, and ≈ 30 ng DNA. PCR amplification was carried out in a T100 Thermal Cycler (BioRad) using the following profile: initial denaturation at 95 °C for 3 min, 32 cycles for 30 s at 95 °C, 1 min at 59 °C, and 1 min/kb at 72 °C. PCR products were stained with SYBR Safe (Invitrogen), separated in a 1.5% agarose gel, and visualized in a ChemiDOC MP system (Biorad).

### Preparation of BAC clones for fluorescence in situ hybridization (FISH)

The full set of BAC clones reported in Damas et al. [15] as suitable for inter-species hybridization in birds were used for hybridization with saker falcon, budgerigar, and ostrich metaphase chromosomes. All experiments were dual color. BAC clone DNA was isolated using the Qiagen Miniprep Kit (Qiagen) prior to amplification and direct labelling by nick translation. Probes were labeled with Texas Red-12-dUTP (Invitrogen) and FITC-Fluorescein-12-UTP (Roche) prior to purification using the Qiagen Nucleotide Removal Kit (Qiagen).

### Cell culture and chromosome preparation

Chromosome preparations were established from fibroblast cell lines generated from collagenase treatment of 5- to 7-day-old embryos or from skin biopsies. Cells were cultured at 40 °C, and 5% CO_2_ in Alpha MEM (Fisher), supplemented with 10% fetal bovine serum (Gibco), 1% Pen Strep/l-glutamine (Sigma). Chromosome suspension preparation followed the standard protocols, and brief mitostatic treatment with colcemid at a final concentration of 5.0 μg/ml for 1 h at 40 °C was followed by hypotonic treatment with 75 mM KCl for 15 min at 37 °C and fixation with 3:1 methanol:acetic acid.

### Fluorescence in situ hybridization (FISH)

Metaphase preparations were fixed to slides and dehydrated through an ethanol series (2 min each in 2 × SSC, 70%, 85%, and 100% ethanol at room temperature). Probes were diluted in a formamide buffer (Cytocell) with Chicken Hybloc (Insight Biotech) and applied to the metaphase preparations on a 37 °C hotplate before sealing with rubber cement. Probe and target DNA were simultaneously denatured on a 75 °C hotplate prior to hybridization in a humidified chamber at 37 °C for 72 h. Slides were washed post-hybridization for 30 s in 2 × SSC w/ 0.05% Tween 20 at room temperature, then counterstained using VECTASHIELD anti-fade medium with DAPI (Vector Labs). Images were captured using an Olympus BX61 epifluorescence microscope with a cooled CCD camera and SmartCapture 3 (Digital Scientific UK) system.

### EBR detection and CNE density analysis

Pairwise synteny blocks were defined using the maf2synteny tool [67] at 100, 300, and 500 kb resolution using the pairwise alignments obtained by lastZ. Using chicken as the reference genome, EBRs were detected and classified using the ad hoc statistical approach described previously [39]. All well-defined (or flanking oriented PCFs) fusion and fission points were identified from pairwise alignments with the chicken genome. Only the EBRs ≤ 100 kb were used for the CNE analysis. EBRs smaller than 1 kb were extended ± 1 kb. For each EBR, we defined two windows upstream (+ 1 and + 2) and two downstream (− 1 and − 2) of the same size as the EBR. We calculated the fraction of bases within CNEs in each EBR site, upstream and downstream windows. Differences in CNE densities were tested for significance using the Kruskall-Wallis test followed by Mann-Whitney *U* test. The CNEs analyzed were identical to those reported in Damas et al. [15].

### Comparing CNE densities in EBRs and msHSBs

Chicken chromosomes (excluding GGA16, W and Z) were divided into 1-kb non-overlapping intervals. Only windows with > 50% of their bases with chicken sequence data available were used in this analysis. All intervals were assigned either to msHSBs > 1.5 Mb [39], avian EBR flanking: fusions, fissions, intrachromosomal EBR, and the intervals found in the rest of the chicken genome. We estimated the average CNE density for each window type and the distance, in number of 1-kb windows, between each window with the lowest CNE density (0 bp) and the nearest window with the average msHSB CNE density or higher. CNE densities were obtained using bedtools (v.2.20-1 [68]). Differences in distances between the two window types in msHSBs and EBRs were tested for significance using the Kruskall-Wallis test followed by Mann-Whitney *U* test. Thus, although the CNEs were the same as in Damas et al. [15], they were analyzed in the context of the new EBRs and mHSBs reported in this study.

## Additional files

Additional file 1: Intrachromosomal rearrangements: BAC IDs and chromosomal orientation of clones (with start and stop coordinates from the chicken genome). The order of clones from the top to the bottom represents the order in which that appears on the chromosomes of the species of interest. Text in red indicates the p- (short) arm of the chromosome (where it is discernable). Data is listed in supplementary tables as follows: **Table S1.** Ostrich genome; **Table S2.** Budgerigar genome; **Table S3.** Saker falcon genome. (ZIP 58 kb)

Additional file 2: Chromosomal coordinates and orientation of mapped scaffolds and PCFs are listed by chromosome for each species. Data is listed in supplementary tables as follows: **Table S4.** Ostrich genome; **Table S5.** Budgerigar genome; **Table S6.** Saker falcon genome. (ZIP 87 kb)

Additional file 3: EBRs detected and genome position in relation to the chicken genome. Data is listed in supplementary tables as follows: **Table S7.** Ostrich genome; **Table S8.** Budgerigar genome; **Table S9.** Saker falcon genome. (ZIP 61 kb)
